# Prevalence of the *dhfr* and *dhps* Mutations among Pregnant Women in Rural Burkina Faso Five Years after the Introduction of Intermittent Preventive Treatment with Sulfadoxine-Pyrimethamine

**DOI:** 10.1371/journal.pone.0137440

**Published:** 2015-09-14

**Authors:** Marc C. Tahita, Halidou Tinto, Annette Erhart, Adama Kazienga, Robert Fitzhenry, Chantal VanOvermeir, Anna Rosanas-Urgell, Jean-Bosco Ouedraogo, Robert T. Guiguemde, Jean-Pierre Van geertruyden, Umberto D’Alessandro

**Affiliations:** 1 Institut de Recherche en Sciences de la Santé/Direction Régionale de l’Ouest (IRSS/DRO), Bobo-Dioulasso, Burkina Faso; 2 Clinical Research Unit of Nanoro (IRSS-CRUN), Nanoro, Burkina Faso; 3 Unité de Recherche sur le Paludisme et Maladies Tropicales Négligées, Centre Muraz, Bobo-Dioulasso, Burkina Faso; 4 Malariology Unit, Institute of Tropical Medicine (ITM), Antwerp, Belgium; 5 International Health Unit, University of Antwerp, Antwerp, Belgium; 6 Institut Supérieur des Sciences de la Santé (INSSA), Bobo Dioulasso, Burkina Faso; 7 London School of Hygiene and Tropical Medicine, London, United Kingdom; 8 Medical Research Council Unit, Banjul, The Gambia; Université Pierre et Marie Curie, FRANCE

## Abstract

**Background:**

The emergence and spread of drug resistance represents one of the biggest challenges for malaria control in endemic regions. Sulfadoxine-pyrimethamine (SP) is currently deployed as intermittent preventive treatment in pregnancy (IPTp) to prevent the adverse effects of malaria on the mother and her offspring. Nevertheless, its efficacy is threatened by SP resistance which can be estimated by the prevalence of dihydropteroate synthase (*dhps*) and dihydrofolate reductase (*dhfr*) mutations. This was measured among pregnant women in the health district of Nanoro, Burkina Faso.

**Methods:**

From June to December 2010, two hundred and fifty six pregnant women in the second and third trimester, attending antenatal care with microscopically confirmed malaria infection were invited to participate, regardless of malaria symptoms. A blood sample was collected on filter paper and analyzed by PCR-RFLP for the alleles 51, 59, 108, 164 in the *pfdhfr* gene and 437, 540 in the *pfdhps* gene.

**Results:**

The genes were successfully genotyped in all but one sample (99.6%; 255/256) for *dhfr* and in 90.2% (231/256) for *dhps*. The *dhfr* C59R and S108N mutations were the most common, with a prevalence of 61.2% (156/255) and 55.7% (142/255), respectively; 12.2% (31/255) samples had also the *dhfr* N51I mutation while the I164L mutation was absent. The *dhps* A437G mutation was found in 34.2% (79/231) isolates, but none of them carried the codon K540E. The prevalence of the *dhfr* double mutations N**RN**I and the triple mutations **IRN**I was 35.7% (91/255) and 11.4% (29/255), respectively.

**Conclusion:**

Though the mutations in the *pfdhfr* and *pfdhps* genes were relatively common, the prevalence of the triple *pfdhfr* mutation was very low, indicating that SP as IPTp is still efficacious in Burkina Faso.

## Background

In Burkina Faso, malaria remains a major cause of morbidity and mortality as it accounts for 60.6% of all hospitalizations and 40.4% of all deaths [1]. *Plasmodium falciparum* is the dominant species, whose transmission is intense and highly seasonal, due mainly to the vector *Anopheles gambiae* [2]. Until 2005, chloroquine (CQ) and sulfadoxine-pyrimethamine (SP) were the first and second line treatment for uncomplicated malaria, respectively. Following reports of CQ and SP treatment failures, the first line treatment was changed to artemether-lumefantrine (AL), with amodiaquine-artesunate (AS-AQ) as an alternative [3]. Nevertheless, SP is still recommended for intermittent preventive treatment during pregnancy (IPTp) in Burkina Faso.

IPTp reduces the prevalence of placental malaria, severe anaemia among primigravidae, pre-term delivery [4, 5], low birth-weight and improves neonatal survival [4–6]. It is considered to be safe, efficacious and easy to administer at antenatal clinics (ANC) [7]. However, in East Africa, resistance to SP administered as IPTp has been associated with an increased risk of foetal anaemia and severe malaria in the offspring [8].

Pyrimethamine selectively inhibits dihydrofolate reductase (*dhfr*), a part of the folate pathway in the malaria parasites, and *Plasmodium falciparum* resistance, both *in vivo* and *in vitro*, has been associated with specific point mutations (A16V, N51I, C59R, S108N/T and I164L) in the *dhfr* gene [9, 10]. Sulfadoxine selectively inhibits dihydropteroate synthetase (*dhps*) earlier in the parasite folate pathway; five point mutations (S436A/F, A437G, K540E, A581G and A613S/T) have been associated with *in vitro* resistance under low or no folate conditions [11, 12]. Increasing SP resistance is associated with an increasing number of mutations in both the *pfdhfr* and *pfdhps* genes; in Africa, the combined *pfdhfr* triple mutant (51I-59R-108N) and the *pfdhps* double mutant (437G-540E), the so called *dhfr/dhps* quintuple mutation, are predictive of SP treatment failure [13]. Therefore, determining the prevalence of these mutations and their evolution over time can provide reasonably good information on temporal trends in SP efficacy. The prevalence of molecular SP resistance markers was determined in asymptomatic and symptomatic malaria-infected pregnant women five years after the introduction of IPTp with SP in Burkina Faso.

## Methods

### Study area, subjects and sample collection

The study was carried out at the Clinical Research Unit in Nanoro (CRUN), situated about 85 km north-west from Ouagadougou. The literacy rate in this area is low for both men and women (about 23%) and there is a high migration flow towards the capital city and/or the neighbouring countries [14]. Malaria transmission is high and seasonal, mainly occurring during the months of August-December, with an entomological inoculation rate (EIR) estimated at 50–60 infective bites/person/year in 2009. The most common vectors are *Anopheles gambiae sensu stricto*, *Anopheles funestus* and *Anopheles arabiensis* (Diabate A., personal communication). Malaria is one of the most common reasons for attending health facilities while *Plasmodium falciparum* is the predominant malaria species [15].

**Fig 1:**
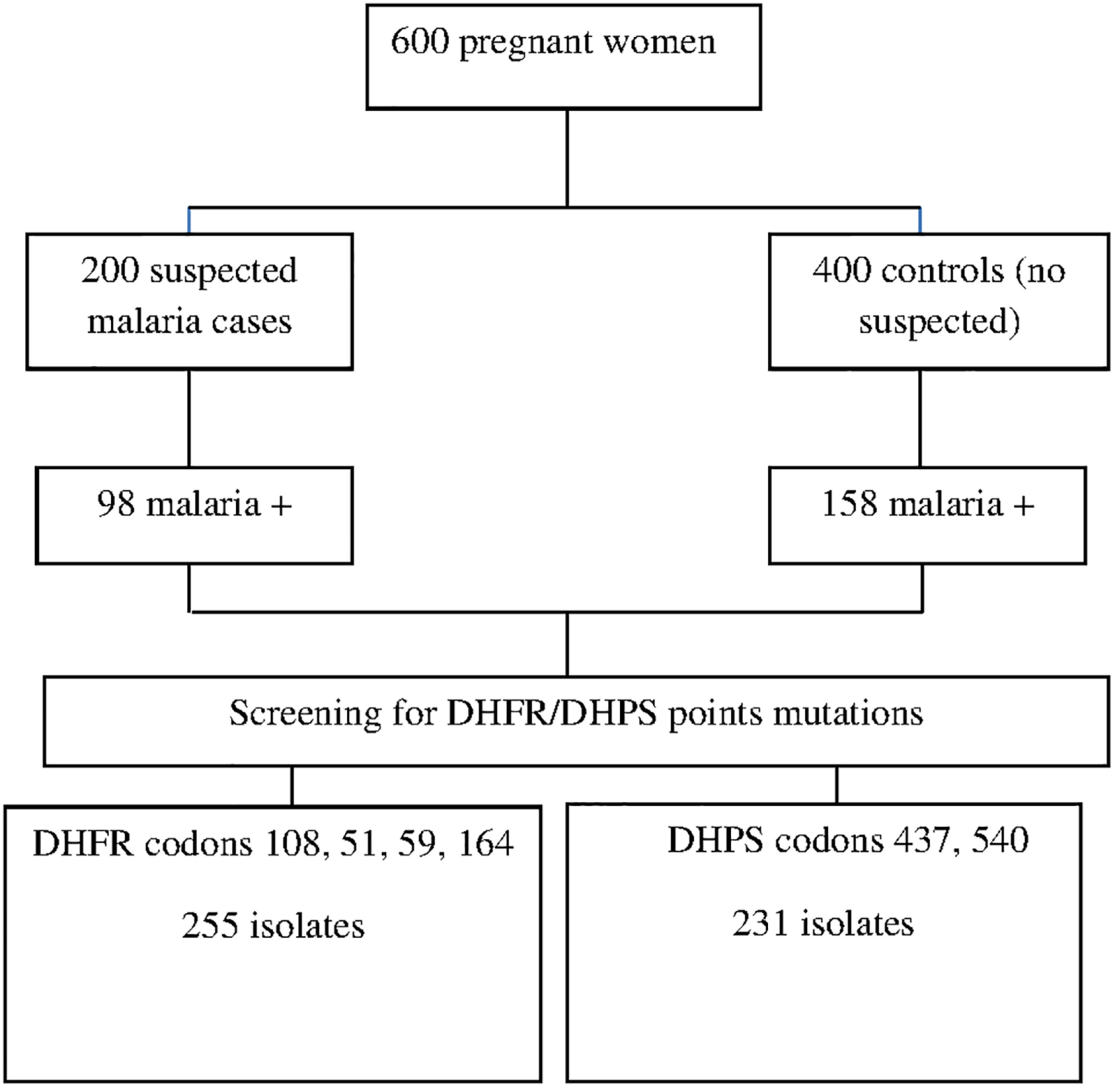
Study profile.

Blood samples were collected as part of a study on the clinical presentation of malaria during pregnancy whose results have been reported elsewhere [16]. Briefly, all pregnant women attending the routine ANC at Nanoro district (Nanoro and Nazoanga health centers) were invited to participate in the study. A finger prick blood sample for slide and dried spots on filter paper (Whatmann grade 3) were collected. Molecular genotyping was performed only on samples from women with a positive blood slide (Fig 1). All molecular tests were performed at the Institute of Tropical Medicine (ITM), Antwerp, Belgium.

### Laboratory procedures

#### DNA extraction

DNA extraction from bloodspots was carried out according to the manufacturer’s instructions using a QIAamp® DNA Micro Kit 50 (Qiagen, Hilden, Germany). Eluted DNA was immediately ready for use in amplification reactions or was stored at –20°C until further processing.

#### Nested PCR and mutation-specific restriction enzyme digestion

The desired molecular products within the *dhfr* and *dhps* genes were amplified by nested PCR following a standardized protocol described elsewhere [17, 18]. The primary *dhfr* amplicon was produced by amplifying sample DNA with primer pairs AMP1 and AMP2 [18]. This PCR product was used in a mutation-specific second PCR reaction to determine the presence of mutations at sites 51, 59, 108 and 164 in the *dhfr* gene. Two separate sets of PCRs were carried out for each codon, one for the wild-type allele and one for the mutant allele.

The HotStarTaq DNA polymerase (Qiagen, Hilden, Germany) was used with the manufacturer’s buffer containing 1.5 mM MgCl2. The primers were used at a concentration of 0.2 mM, and other reaction conditions were as described previously [18] with the following cycling parameters: initial denaturation at 95°C for 5 min, followed by 35 cycles of denaturation at 92°C for 1 min, annealing at 52°C for 45 sec, extension at 72°C for 45 sec, and a final extension at 72°C for 10 min. Screening for *dhps* mutations was carried out as for *dhfr* screening with the following modifications. DNA was amplified using primers DPHS-R1 and DHPS-R2. This primary amplification product was subjected to a second round diagnostic PCR as part of a nested PCR, with the primers DHPS-K and DHPS-K1, followed by restriction enzyme digestion [17]. Digestion products were analyzed on a 2% agarose gel with ethidium bromide.

For all *dhfr* codons, the *P*. *falciparum* strain 3D7 was used as wild-type control and V1S as mutant control. For the detection of *dhps* mutations, PS-Mali-clone DNA and PS-Peru-clone DNA were used as wild-type and mutant control for positions 437 and 540, respectively. The presence of *dhps* mutations A437G was evaluated by digestion with AvaII, and of *dhps* K540E mutation by digestion with FokI, both enzymes cleaving the mutated sequences.

### Statistical methods

Data were entered in Excel version 2007 and analyzed using STATA v10 (STATA Corporation, College Station, TX, USA). In this analysis, mixed genotypes were considered as mutants, and the prevalence of each type of allele (wild or mutant) were calculated together with their respective confidence intervals. Participants were categorized as symptomatic and asymptomatic. Symptomatic women were defined as women having at least one of the following signs and symptoms: temperature ≥37.5°C (measured by electronic thermometer) and/or history of fever in the previous 48 hours, headache, pallor, arthro-myalgia, convulsions, vomiting, dizziness, malaise, fatigue, enlarged liver or enlarged spleen. Asymptomatic women had none of the above mentioned symptoms.

The frequencies of the mutations were compared between these groups, parasite density, and age using chi-square test, and a p-value <0.05 was considered as statistically significant.

### Ethical considerations

After reviewing the study protocol, the Institutional Ethics Committee of the Centre Muraz, Bobo-Dioulasso, Burkina Faso (registration no. 005-2010/CE-CM) approved the study. Participants were only included after obtaining their written informed consent.

## Results

Six hundred pregnant women were included in the study, of whom two hundred and fifty six (42.7%) had a microscopically-confirmed malaria infection (Fig 1). Most of them (81%) were aged 20–34 years old and had already one to three children (85%). Parasite density was not associated with the occurrence of symptoms (Table 1).

**Table 1:** Baseline characteristics by presence or not of symptoms.

	Asymptomatic	Symptomatic	p-value
	n = 157	n = 198	
**Age group (years)**			
<20	13(8.3%)	11(11.2%)	0.66
20–34	128(81.5%	79(80.6%)	
≥35	16(10.2%)	8(8.2%)	
**Parasite density**	710.61(541.45–932.62)	1107.46(747.48–640.82)	0.08
**Parity**			
Nulliparous	13(8.3%)	10(10.2%)	0.77
1 to 3	101(64.3%)	59(60.2%)	
≥4	43(27.3%)	29(29.6%)	
**Anemia**	95(60.5%)	75(76.5%)	0.01
**IPTp(doses received)**			
0	47(30%)	28(28.6%)	077
1	90(57.3%)	60(61.2%)	
2	20(12.7%)	10(10.2%)	

Most samples could be successfully genotyped, 99.6% (255/256) for the *dhfr* gene and 90.2% (231/256) for the *dhps* gene (Fig 1). More than half of the samples had the *dhfr* C59R (61.2%, 156/255) and/or the S108N (55.7%, 142/255) mutations while only 12.2% (31/255) had the N51I mutation, and no I164L mutation was found (Table 2).

**Table 2:** Prevalence of the *dhfr* and *dhps* point mutations associated with SP resistance.

	*dhfr* (N = 255)				*dhps* (N = 231)	
**Codon**	**51**	**59**	**108**	**164**	**437**	**540**
**Mutant, n(%)**	31(12.2)	156(61.2)	142(55.7)	0	79(34.2)	0
**[95% CI]**	[8.7–16.7]	[55.1–67.0]	[49.5–61.7]	-	[28.4–40.5]	-

There were 6 different *dhfr* alleles; the prevalence of the sequence NCSI (wild type) was 30.2% (77/255). Among the mutant alleles, the double mutation N**RN**I was the most frequent (35.7%, 91/255), followed by the triple mutation **IRN**I (11.4%, 29/255) (Table 3).

**Table 3:** *Dhfr* mutations among isolates.

	NCSI	ICSI	NRSI	NCNI	NRNI	IRSI	ICNI	IRNI
**51**								
**59**								
**108**								
**164**								
**n**	77	0	34	22	91	2	0	29
**%(x/255)**	30.2	0	13.3	8.6	35.7	0.8	0	11.4
**95%CI**	24.8–36.2	-	9.7–18.1	5.7–12.7	30.1–41.7	0.2–2.8	-	8.1–15.9

More than a third of the samples (34.2%, 79/231) carried the *dhps* mutations A437G but none of them had the mutation K540E.

The occurrence of the mutations N51I and A437G were significantly associated with higher parasite density (Table 4). No other factor (age, parity and number of SP doses taken) was found to be associated with the risk of double or triple mutation.

**Table 4:** Trends of the molecular markers according to the parasite density.

	Parasite density(geometric mean)		**P-value**
	Symptomatic	Asymptomatic	
***dhfr* 51**	12558.38	3970.26	0.02
	(5078.04–31057.85)	(2391.12–6592.31)	
***dhfr* 59**	1419.81	1076.25	0.37
	(841.74–2394.88)	(754.03–1536.17)	
***dhfr* 108**	1576.97	1454.58	0.80
	(890.82–2791.60)	(1024.57–2065.06)	
***dhps* 437**	1873.07	708.17	0.01
	(989.73–3544.78)	(443.41–1131.01)	

## Discussion

Despite several studies on the association between genetic polymorphisms and response to SP treatment, the role of certain *dhfr* and *dhps* mutations in treatment outcome is still poorly understood. SP resistance increases with the increasing number of point mutations in the *dhfr* and *dhps* genes [19]. In Nanoro, among pregnant women, the most prevalent *dhfr* allele’s mutations were 59R and the 108N, as more than half of the isolates carried one of them. However, the prevalence of the double mutation 59R and 108N was much lower, about 36%, and the triple mutation 108N-51I-59R had an even lower prevalence, around 11%. Though single or double mutations in the *dhfr* gene have been associated with pyrimethamine resistance [20–23], the *dhfr* triple mutation is known to confer intense pyrimethamine resistance *in vitro* [24] and is associated with an approximate 1,000-fold reduction in pyrimethamine susceptibility [25]. Nevertheless, SP is systematically administered to all pregnant women in the second and third trimester attending ANC, while only a proportion of them would carry a malaria infection, often of low density. For other non-infected pregnant women at the time of treatment, SP would have a prophylactic effect as it would clear emerging malaria infections for a given period of time. The low prevalence of the triple *dhfr* mutations indicates that in Nanoro SP should be able to clear malaria infections present at the time of its administration, particularly when considering that their density would be generally low. The effect of the *dhfr* double or triple mutation on the duration of the protection period is unknown but its duration may be shorter and parasites carrying the double or triple mutation may be able to emerge earlier than the wild ones.

No isolate had the *dhps* double mutation, at position 437 and 540, commonly associates with sulfadoxine resistance [26, 27]. Indeed, in more than a third of all isolates it was possible to identify only the 437 mutation, which usually occurs first in the progressive selection of resistant parasites [28]. Such mutation, alone or combined with the K540E, has been associated with treatment failure with SP [27, 29, 30]. Nevertheless, in Nanoro, the A437G prevalence was low compared to that found in north western Burkina Faso where in the year 2000 the prevalence was already above 50% and had increased to almost 80% in 2009 [31].

The low prevalence of the triple *dhfr* mutation is surprising when considering that SP was extensively used as first line treatment in Burkina Faso and that in other areas of Burkina Faso it was significantly higher [32], with suggestion of its increase over time [31]. Such discrepancy may be due to differences in drug pressure and differing access to ACT treatment. Indeed, before 2005, SP was used as second line treatment and after 2005 as first line treatment, before the large scale implementation of ACT. The remaining high prevalence of *dhfr* triple mutants could indicate a less than optimal access to ACT in Ziniare [32] and Nouna [31], while in Nanoro the ready access to ACT could have resulted in a decreased prevalence of the triple mutants. Such phenomenon has already been observed in South America where a significant decline of the prevalence of *dhfr* triple mutants coincided with the change of the treatment policy from SP to ACT [33, 34], suggesting a lower fitness of SP-resistant parasites in the absence of a significant drug pressure. Nevertheless, in Malawi the prevalence of mutant parasites did not decrease after five years without SP as first line treatment, indicating no fitness cost of the *dhfr* triple and *dhps* double mutant haplotypes in the absence of strong SP pressure [35].

None of the isolates carried the *dhps* K540E mutation while more than one third had the A437G mutation. This is not surprising when considering that none of the recent studies carried out in Burkina Faso found isolates with the K540E mutation [31, 32]. Nevertheless, these same studies reported a much higher prevalence of the A437G mutation in other parts of the country, over 70%, possibly indicating a much higher drug pressure compared to Nanoro.

Infections carrying one or more of the *dhfr* mutations were associated to higher parasite density together with the presence of symptoms. This was true for the *dhfr* 51 and *dhps* 437 mutations. This could have major implications for the severity of the disease and for selection pressure as these mutations do not seem to confer a survival disadvantage for the parasite.

## Conclusion

Mutations in the *Pfdhfr* and *Pfdhps* genes associated with SP resistance were relatively common among pregnant women in the study area. Nevertheless, the prevalence of the triple *dhfr* mutations was very low suggesting that SP may still be efficacious when used as IPTp. Nevertheless, molecular markers linked to SP resistance should continue to be monitored.
